# Streptococcal necrotising fasciitis from diverse strains of *Streptococcus pyogenes *in tropical northern Australia: case series and comparison with the literature

**DOI:** 10.1186/1471-2334-4-60

**Published:** 2004-12-16

**Authors:** Marilyn Hassell, Peter Fagan, Phillip Carson, Bart J Currie

**Affiliations:** 1 Infectious Diseases Unit, Northern Territory Clinical School, Flinders University, Royal Darwin Hospital, Darwin Northern Territory Australia; 2 Infectious Diseases Division, Menzies School of Health Research, Charles Darwin University, Darwin Northern Territory Australia; 3 Department of Surgery, Northern Territory Clinical School, Flinders University, Royal Darwin Hospital, Darwin Northern Territory Australia

## Abstract

**Background:**

Since the mid-1980's there has been a worldwide resurgence of severe disease from group A streptococcus (GAS), with clonal clusters implicated in Europe and the United States. However GAS associated sepsis and rheumatic fever have always remained at high levels in many less developed countries. In this context we aimed to study GAS necrotising fasciitis (NF) in a region where there are high background rates of GAS carriage and disease.

**Methods:**

We describe the epidemiology, clinical and laboratory features of 14 consecutive cases of GAS NF treated over a seven year period from tropical northern Australia.

**Results:**

Incidence rates of GAS NF in the Aboriginal population were up to five times those previously published from other countries. Clinical features were similar to those described elsewhere, with 7/14 (50%) bacteremic and 9/14 (64%) having associated streptococcal toxic shock syndrome. 11/14 (79%) had underlying chronic illnesses, including all four fatalities (29% mortality overall). Important laboratory differences from other series were that leukocytosis was absent in 9/14 (64%) but all had substantial lymphopenia. Sequence typing of the 14 NF-associated GAS isolates showed no clonality, with only one *emm *type 1 and two *emm *type 3 strains.

**Conclusions:**

While NF clusters can occur from a single emergent GAS clone, this was not evident in our tropical region, where high rates of NF parallel high overall rates of GAS infection from a wide diversity of strains. The specific virulence factors of GAS strains which do cause NF and the basis of the inadequate host response in those patients who develop NF on infection with these GAS require further elucidation.

## Background

Necrotising fasciitis (NF) is an aggressive and rapidly destructive soft tissue infection resulting in high mortality and significant long term morbidity. Two groups of infectious agents have been described – Type I which are polymicrobial, involving anaerobic bacteria and streptococci other than serogroup A, and Type II from *Streptococcus pyogenes *(Group A streptococci, GAS) alone, or in association with *Staphylococcus aureus *or *Staphylococcus epidermidis *[1]. The latter was originally described by Meleney in 1924 as 'haemolytic streptococcal gangrene' [2] and has since been considered a rare entity.

Since the mid-1980's a worldwide increase in infections due to GAS has been noted. In affluent populations where GAS disease is uncommon aside from pharyngitis in childhood, increasing numbers of necrotising fasciitis and streptococcal toxic shock syndrome (STSS) have been seen, as well as an upsurge of acute rheumatic fever apparently restricted to parts of the United States [3-6]. This has been attributed in some locations to dissemination of a virulent M1 serotype GAS clone[7]. In developing nations the pattern of GAS disease is different, with continuing high rates of streptococcal pyoderma and post-streptococcal disease[8].

Recent case series of GAS NF have been published from Norway[4] and Ontario, Canada[5,6]. The Northern Territory (NT) of Australia consists of a combination of affluent, urban residents, as well as residents in remote communities, who are predominantly indigenous Aboriginals with high levels of poverty and overcrowding. As in developing nations, these remote communities have very high rates of GAS disease in the form of streptococcal pyoderma, rheumatic fever and post-streptococcal glomerulonephritis[9]. We examine the epidemiology, clinical features and streptococcal sequence typing of a series of cases of GAS NF from the tropical top end of the NT and compare the results with the literature.

## Methods

The Royal Darwin Hospital services 135,000 inhabitants of the NT, with around 100,000 located in the city of Darwin. Indigenous Aboriginal Australians account for 24% of the population[10]. Cases of possible GAS NF treated at Royal Darwin Hospital were prospectively identified. Case definition for inclusion in the analysis required GAS to be cultured from sterile sites, notably operative tissue specimens +/- blood cultures. NF was defined by necrosis of subcutaneous tissue, specifically fascial oedema and necrosis detected either macroscopically at surgery, or microscopically on histopathology.

Fourteen cases fulfilling the case definition were identified between May 1994 and April 2001. A chart review was conducted to identify epidemiological characteristics, clinical features, laboratory results, treatment and outcomes. Age, gender, ethnicity, urban or rural residence and any concurrent medical conditions were documented. The dates of presentation, presumptive diagnosis of NF and of surgery were documented. Symptoms, signs, hemodynamic status and temperature were documented for these dates, as was the presence of STSS as defined by the Working Group on Severe Streptococcal Infections[11]. Antibiotic treatment, surgery, and any adjuvant therapies were documented. Baseline hematological and biochemical parameters were recorded for the date of presentation, and blood and tissue culture results were documented. Duration of Intensive Care Unit (ICU) admission, and type of ICU support required were recorded.

Incidence rates of GAS NF for this region of the NT were calculated using regional population numbers from census data. There was possibly an increase in case ascertainment after 1998 and incidence rates were also calculated by year.

Procedures for *emm *sequence typing and analysis of *emm*-specific PCR products were carried out as previously described[12]. DNA sequences were subject to homology searches against all *emm *sequences deposited in GenBank and in the Centers for Disease Control (CDC) *Streptococcus pyogenes emm *sequence database[13].

Approval for the study was obtained from the Health Research Ethics Committee of Royal Darwin Hospital and the Menzies School of Health Research.

## Results

Table 1 summarizes the details for each patient.

From 1994 to 2001, 14 cases of confirmed GAS NF were treated at Royal Darwin Hospital. The number of cases per year ranged from zero in 1995 and 1998 to five in 2001. This gives a maximum yearly incidence of 3.8 cases per 100,000. Half of the cases occurred in Aboriginal patients, with an incidence of 5.8 per 100,000 for Aboriginals in the 2001 cluster of cases. Eight cases occurred in women (57%), five of whom were Aboriginal. Ages ranged from 27–61 years in males (mean 41.5 yrs) and 27–59 years in females (mean 41.5 yrs). Eleven cases were community acquired and three nosocomial. All seven Aboriginal patients had multiple concurrent medical conditions, whereas three of the seven non-Aboriginals had no additional pathology.

Patients presented most consistently with pain in the affected area. The other common complaints were swelling, and systemic symptoms of fever and rigors. Signs on presentation were typically induration and tense swelling of the area. Only four of the fourteen cases (29%) were noted to have extensive skin erythema, but nine (64%) had local pre-existing wounds or ulceration. Ten patients had only limb involvement, two had chest wall involvement, one had extension from right leg to the abdominal wall and one had extension from the left buttock to perineum/labia. STSS occurred in nine patients. Ten patients required inotropic support, six had a documented coagulopathy and eleven had renal impairment. Thirteen patients (93%) required ICU stay of 1–24 days. Eleven patients were ventilated, and six received renal replacement therapy.

Total white cell count (WCC) was found to be raised (>11 × 10^9^/L) in only 5/14 (36%) patients, although lymphocyte count was globally reduced, being <1.0 × 10^9^/L in all patients, and <0.5#215;10^9^/l in nine cases (64%). Renal function was frequently impaired, with 11/14 (79%) patients having raised urea (mean 17.7 mmol/L, NR 3.0–8.0 mmol/L) and 12/14 (86%) having raised creatinine (mean 301 μmol/L, NR 50–100 μmol/L). All patients were hypoalbuminemic with 11 having albumin <30 g/L (mean 24.5, range 11–34, NR 35–45 g/L) Plasma creatine kinase (CK) ranged from 25 U/L to 2526 U/L (NR <220 U/L), being elevated in 5/9 (56%) patients measured. C-Reactive Protein (CRP) was substantially raised in all 11 where measured, being between 108–522 mg/L (NR<10). Blood cultures were positive for GAS in seven cases (50%). GAS was isolated from all operative tissue specimens, with four also positive for *S. aureus*.

One GAS isolate could not be sequenced. GAS from the other 13 patients showed a large diversity of *emm *sequence types, with no clonality. Where blood and tissue GAS were both recovered from an individual, the *emm *sequence types were the same. One patient's GAS isolate was a previously uncharacterized *emm *sequence type and there was only one case with *emm *sequence type 1 GAS isolated. The two *emm *3 isolates were from the 2001 cluster of five cases. However they were very divergent on random amplified polymorphic DNA analysis (data not shown), one being *emm *sequence type 3.2, and the other three isolates in this cluster were all different *emm *types.

All patients underwent surgery 0–7 days after presentation. Debridement alone was conducted in nine patients, and combined with fasciotomy in one case. Fasciotomy alone was done in one case. Three patients underwent limb amputation. At surgery it was established that three patients (21%) had concurrent myonecrosis. Antibiotic therapy was instituted with a beta lactam in all patients (benzylpenicillin in nine, meropenem in three, cephazolin in one and flucloxacillin in one). Clindamycin was also given in eleven patients. Adjuvant hyperbaric oxygen therapy was given in four cases, and intravenous immunoglobulin (IVIG) in one.

Four patients (29%) died, all being Aboriginal females with co-morbidities. Two of the four patients with truncal infection died in comparison to only two of the ten with only peripheral involvement. The mean time to surgery from presentation was 4.25 days in patients who died and 3.7 days in survivors (not significant). Blood cultures were positive in 3/4 fatalities with STSS in 3/4. All four were in ICU for 1–5 days until death, with all requiring inotropes and mechanical ventilation, two given renal replacement therapy and one IVIG. Two received clindamycin in addition to beta-lactam antibiotic therapy. Surgery in the fatal cases consisted of debridement and/or fasciotomy, with no amputation.

## Discussion

### Demographics

We describe a series of 14 cases of GAS necrotising fasciitis in a region with a high background incidence of GAS disease[9]. Our incidence of NF in 2001 of 3.8/100,000 and 5.8/100,000 in the Aboriginal population is higher than previously published rates which range from 0.4/100,000 in Canada[5] to 2/100,000/yr in Norway [4]. However the population denominators in the Canadian and Norwegian studies are far greater than in our study. Nevertheless, previous data from our region showed that rates of GAS bacteremia in the Aboriginal population were five times those in the Caucasian population[9]. Therefore NF in our population parallels an overall greater rate of GAS infection, most notably in the Indigenous population where conditions reflect those of developing countries[8].

The mean age of our patients (41.5 years) is lower than other reports showing an average age of 56–58 years, with notable increases in incidence with increasing age[4-6]. Unlike all other series which had a male predominance, 57% of our cases were female, with 71% females amongst the Aboriginal cases. The age and sex pattern seen in our series reflects the high rates of chronic diseases in the Indigenous population, with mortality from all causes being higher in all age cohorts, but especially so in females[14,15]. 79% of our patients had concurrent chronic disease, compared with 46–71% in previous series[4-6,16]. In this series 64% had pre-existing wounds, consistent with reported rates of 47–66%[4-6,16]. None of our patients had preceding varicella infection.

### Laboratory results

Two studies have looked at the value of early blood test results in predicting NF as opposed to non-NF soft tissue infection. One found that CK >600 U/L achieved PPV of 58% for NF[17]. However only 1/9 cases in this series had CK >600 U/L. CRP of >16 mg/L was also thought to be indicative of NF[16]. CRP was over 100 in all those measured in our series. Raised WCC has also been suggested as useful in a predictive model of early NF[18], with rates of 66–73% in other series[4-6]. However, only 36% of our patients had a raised WCC. We found lymphopenia to be a more consistent factor, with 100% of patients having a lymphocyte count of <1.0 × 10^9^/L, 64% of those being <0.5 × 10^9^/L. Low albumin was also universal.

### Clinical features

STSS occurred in 64% of our cases in comparison to 40–46% in other series[4-6]. 71% of our patients were hypotensive at presentation, with 86% having renal impairment in comparison to 35–61% in other series. Similarly to the other series, 93% of our patients were admitted to the ICU but more of our patients required mechanical ventilation (79%) and inotropic support (71%). Positive blood cultures are not a prerequisite for GAS NF, as stated by Meleney in the initial descriptions[2], and reinforced in subsequent studies showing bacteremic rates of 38–60%[4-6,16], consistent with 50% in this study.

Case-fatality rates for NF previously reached 50%, with mortality of 80–100% in GAS myositis[3]. Recent series report lower case-fatality rates of 20–34%[4-6], with our mortality being 28%. As in previous studies, deaths were more common in those with underlying illness, truncal infection, bacteremia, STSS, myonecrosis and delay in diagnosis and appropriate therapy.

### Therapy

Immediate, extensive surgical debridement of all necrotic tissue is considered essential for optimal treatment of NF, with one early observational study showing a mortality of 0% with aggressive early surgery in comparison to 50% in historical controls[19,20]. Surgery was conducted on the day of presumptive diagnosis of NF in all our patients, but none of the four fatal cases underwent amputation and one with upper limb involvement had only a fasciotomy performed.

Combination therapy with benzylpenicillin and clindamycin is now recommended treatment for proven GAS NF and STSS[21-23]. Experimental data suggest that penicillin is not as bactericidal when there are large numbers of GAS organisms present[24], with decreased expression of penicillin binding proteins when large inocula reach a stationary growth phase[25]. The potential benefits of clindamycin have been supported by a murine model of streptococcal myositis[26]. Clindamycin is a protein synthesis inhibitor, potentially also suppressing bacterial toxin and M protein production[25]. Furthermore, clindamycin may modulate the host immune response, by reducing lipopolysaccharide-induced monocyte production of TNFα[27]. A combination of β-lactam antibiotic and clindamycin has been shown to be superior to β-lactams alone in a retrospective review of invasive GAS infection in 56 children[28]. A recent retrospective review of notified invasive GAS infections in Florida, USA showed the use of clindamycin to be associated with lower mortality in NF cases, but not in other invasive GAS infections[29]. However there have been no randomised clinical trials.

Hyperbaric oxygen (HBO) therapy has been advocated as adjuvant therapy for both microbiological types of NF. Although there are no randomised trials, NF is an approved Undersea and Hyperbaric Medical Society indication for HBO therapy[30]. It is suggested that increased tissue oxygen partial pressures increases bacterial killing by increased respiratory burst and increased formation of oxygen free radicals. HBO is postulated to facilitate wound healing by supporting fibroblast proliferation and angiogenesis[30,31]. In a murine model of GAS myositis, the combination of penicillin and HBO therapy exerted at least additive effects in decreasing bacterial counts in vivo and increasing survival[32]. An observational study of 29 patients with NF showed significantly lower mortality, and need for fewer debridements in those receiving adjunctive HBO, despite the HBO group being more seriously ill[33]. Patients with non-clostridial fasciitis appeared to have greater benefit. However more recently a retrospective evaluation of 37 patients treated for NF showed higher mortality with greater need for debridement in those receiving HBO[34].

The clinical picture of STSS has been attributed to superantigens produced by GAS, including pyrogenic exotoxins, streptococcal superantigen SSA and a mitogenic exotoxin[35]. This is supported by selective depletion of Vβ-bearing T cells in patients with STSS[36]. However GAS isolates from both severe and uncomplicated disease can produce large amounts of toxins. Furthermore, in comparison to patients with uncomplicated infection the sera of patients with severe GAS disease had low antibody levels against erythrogenic toxins[37], suggesting an important role for host humoral immunity. IVIG has therefore been proposed as therapy to increase neutralizing antibody levels in patients with severe GAS disease. A study of 12 patients, 11 with STSS and one with GAS NF, showed increased capacity of plasma to neutralize superantigenic activity following IVIG[38]. Post-IVIG plasma from each patient completely blocked cytokine production elicited by their respective GAS culture supernatants or by purified streptococcal pyrogenic exotoxins. GAS superantigens and cytokines suggestive of superantigen response have been isolated from tissue samples in patients with GAS NF[39]. More severe clinical disease correlated with significantly higher bacterial load in biopsy samples, and bacterial load in turn correlated with expression of superantigenic toxins. This supports a role for IVIG in GAS NF, with or without STSS. Adjunctive IVIG therapy in STSS was evaluated in a multicenter, randomized, double-blind, placebo-controlled trial[40]. Although prematurely terminated due to slow patient recruitment, there was a non-significant 3.6 fold higher mortality rate in the placebo group and a significant decrease in sepsis-related organ failure assessments on days two and three in the IVIG group. There are case reports and two case series of four and seven patients describing clinical improvements of patients with STSS following IVIG[41-43]. In a series of 20 cases of GAS NF and myonecrosis, 16 were treated with IVIG[6]. Although the case fatality rate was not significantly lower in those receiving IVIG, the overall survival rates of 80% in patients with NF and 63% in patients with myonecrosis were much higher than previously reported[6].

In summary, there is in vitro and in vivo experimental data to suggest a benefit for clindamycin, HBO therapy and IVIG in GAS NF and myositis. This is supported by limited case series but not by randomized controlled trials. The primary role of early, aggressive surgery in GAS NF remains probably the most critical factor in minimizing mortality. Clindamycin is recommended for GAS NF but whether clindamycin alone is as effective as penicillin plus clindamycin remains unclear. Benefit from HBO remains unproven, while there is emerging evidence for the use of IVIG, especially if STSS is present.

### Molecular epidemiology

Following a reported resurgence in invasive GAS infections in the 1980's, epidemiological studies proposed that dissemination of a new, more virulent strain of M1 serotype GAS was the cause for a large proportion of these infections[7]. In reports from the United States, Europe and New Zealand the proportion of this strain appeared to increase, both in those with uncomplicated pharyngitis and in invasive GAS infections[37,44-47]. The M1 serotype has also been reported to have a stronger association with STSS[48]. In contrast to these findings, other groups have noted a significant diversity amongst the organisms causing severe streptococcal infections, as well as significant clinical variation amongst patients infected with identical organism clones, suggesting that the severe infections are not related to one specific virulent clone[49-51]. Our findings are consistent with the latter, with GAS sequence typing showing all NF isolates to be different and only 1/13 to be *emm *type 1. This reflects previous findings of enormous diversity amongst organisms causing GAS bacteremia in our region, with no evidence of a dominant clone[9]. As well as finding no correlation between NF and specific M types, a recent Dutch study also found that no specific toxin genes or genes encoding matrix binding proteins were associated with NF[48].

## Conclusions

In conclusion we have described a case series of necrotising fasciitis due to GAS for a unique mixed population of patients living in both affluent and disadvantaged society. Our overall incidence rates of GAS NF are higher than previously reported, with rates in the Aboriginal population up to five times those previously published. The majority of those with NF had underlying chronic illnesses and mortality was also higher in this group. Important laboratory findings were that leukocytosis was absent in more than half of cases but all had substantial lymphopenia. *Emm *sequencing typing of GAS isolates showed none to be the same and only 1/13 to be *emm*1. GAS NF in our region is not clonal but occurs from a wide diversity of GAS strains that reflect the very high background rate of both uncomplicated GAS infection and invasive disease. While NF case clusters can occur from a single emergent GAS clone, this is not evident in our tropical region. The specific virulence factors of the GAS which do cause NF and the basis of the inadequate host response in those patients who develop NF on infection with these GAS require further elucidation.

## Competing interests

The authors declare they have no competing interests.

## Authors' contributions

MH collated and analysed the case data and was principal writer of the manuscript, PF carried out the molecular studies, PC participated in the study design and patient management, BC conceived the study and participated in the study design and coordination and patient management. All authors read and approved the final manuscript.

## Pre-publication history

The pre-publication history for this paper can be accessed here:



## Figures and Tables

**Table 1:** Demographic, clinical and laboratory details of 14 patients with GAS necrotising fasciitis

**No**	**Age/Gender/Aboriginal/Year**	**Co-morbidities**	**Prior lesions**	**Systolic BP at presenttion (mmHg)**	**Creatinine (μmol/L) (NR 50–100)**	**Blood culture**	***emmST* ^a^**	**STSS^b^**
1	45/F/Y/2001	Alcohol excess	Right knee abrasion	85	810	Neg	new *emmST*	Y
2	50/F/Y/1994	Rheumatoid arthritis	Nil	100	352	Pos	*emm*56	N
3	47/F/Y/2000	Hemodialysis, Hypertension	Nil	85	739	Pos	*emm*ST3757	Y
4	38/F/Y/2001	Type 2 diabetes, Hemodialysis, Cryptococcal pneumonitis	Buttock ulcer	60	408	Pos	no sequence	Y
5	61/M/N/2001	Rheumatoid arthritis, Corticosteroid therapy, Splenectomy	Vasculitic leg ulcer	70	249	Pos	*emm*3.2	Y
6	60/M/N/2001	Rheumatoid arthritis, Methotrexate therapy, Pulmonary fibrosis	Leg ulcer	90	237	Neg	*emm*70	Y
7	27/M/N/1996	Alcohol excess	Nil	70	172	Neg	*emm*58	Y
8	34/F/Y/1996	SLE, Anti-Phospholipid syndrome, Corticosteroids, Renal impairment	Left thigh skin sore	100	329	Pos	*emm*69	N
9	44/M/Y/2000	Type 2 diabetes, Alcohol excess, Renal impairment	Wound from catfish barb	90	271	Neg	*emm*49.3	Y
10	27/F/N/1999	Nil	Nil	90	77	Neg	*emm*104	N
11	32/F/N/1999	Nil	Buttock excoriation post-cycling	100	177	Neg	*emm*1	N
12	59/F/N/1999	Nil	Nil	90	118	Neg	*emm*22	Y
13	50/M/Y/1997	Alcohol excess, Cirrhosis	Leg ulceration	100	112	Pos	*emm*4	N
14	61/M/N/2001	Alcohol excess, Hypertension, Gout	Ulcerated gouty tophus	90	659	Pos	*emm*3	Y
**No**	**WCC/Lymph^c ^(×10^9^/L)**	**Albumin (g/L) (NR 35–45)**	**Infection site**	**Time to surgery after presentation**	**Operation performed**	**Antibiotic therapy**	**Myo-necrosis**	**Died**

1	5.7/0.2	21	Right leg	4 days	Debridement, Fasciotomy	Meropenem, Clindamycin	Yes	Yes
2	5.2/0.5	22	Right arm	6 days	Fasciotomy	Penicillin	Yes	Yes
3	9.6/0.3	33	Right breast/Chest wall	5 days	Debridement	Penicillin	No	Yes
4	9.3/0.4	34	Left leg/Abdominal wall	2 days	Debridement	Penicillin, Clindamycin	No	Yes
5	36.7/0.7	23	Left leg	1 day	Above knee amputation	Meropenem, Tobramycin, Clindamycin	No	No
6	23.7/0.5	29	Right leg	0 days	Below knee amputation	Cefazolin, Clindamycin	No	No
7	5.2/0.1	25	Right leg	5 days	Debridement	Penicillin, Clindamycin	No	No
8	7.0/0.3	33	Left leg/Abdominal wall	7 days	Debridement	Penicillin, Clindamycin	Yes	No
9	22.8/0.8	11	Right foot	2 days	Above knee amputation	Flucloxacillin, Metronidazole, Gentamicin	Yes	No
10	23.3/1.0	29	Left shoulder/Chest wall	6 days	Debridement	Meropenem, Clindamycin	No	No
11	21.8/0.5	17	Right leg/Buttock/Perineum	2 days	Debridement	Penicillin, Clindamycin	No	No
12	4.8/0.4	18	Left leg/Abdominal wall	2 days	Debridement	Penicillin, Clindamycin	No	No
13	10.5/0.7	21	Left leg	5 days	Debridement	Penicillin, Clindamycin	No	No
14	4.0/0.2	28	Right leg	4 days	Above knee amputation	Penicillin, Clindamycin	No	No

^a ^*Emm *sequence type (see methods for details)

^b ^Streptococcal toxic shock syndrome (see methods for details)

^c ^WCC-total blood white cell count (normal range <11.0 × 10^9^/L) Lymph-blood lymphocyte count
